# Impact of Ball Burnished Regular Reliefs on Fatigue Life of AISI 304 and 316L Austenitic Stainless Steels

**DOI:** 10.3390/ma14102529

**Published:** 2021-05-13

**Authors:** Stoyan Slavov, Diyan Dimitrov, Mariya Konsulova-Bakalova, Dimka Vasileva

**Affiliations:** 1 Department of Mechanical Engineering and Machine Tools, Technical University of Varna, 9010 Varna, Bulgaria; mbakalova@tu-varna.bg (M.K.-B.); d.vasileva@tu-varna.bg (D.V.); 2 Department of Mechanics and Machine Elements, Technical University of Varna, 9010 Varna, Bulgaria; dm_dimitrov@tu-varna.bg

**Keywords:** ball burnishing, regular reliefs, surface topography, austenitic stainless steel, fatigue life, *t*-test, Bayesian rule

## Abstract

The present work describes an experimental investigation of the fatigue durability of AISI 304 and AISI 316L austenitic stainless steels, which have regular reliefs (RR) of the IV-th type, formed by ball burnishing (BB) on flat surfaces, using a computer numerical control (CNC) milling center. The methodology and the equipment used for obtaining regular reliefs, along with a vibration-induced fatigue test setup, are presented and described. The results from the BB process and the fatigue life experiments of the tested austenitic stainless steels are gathered, using the approach of factorial design experiments. It was found that the presence of RR of the IV-th type do not worsen the fatigue strength of the studied steels. The Pareto, *t*-test and Bayesian rule techniques are used to determine the main effects and the interactions of significance between ball burnishing regime parameters. A stochastic model is derived and is used to find when the probability of obtaining the maximum fatigue life of parts made of AISI 304 or 316L reaches its maximum value. It was found that when the deforming force, the amplitude of the sinewaves and their wavenumber are set at high values, and the feed rate is set at its low value, the probability to reach maximum fatigue life for the parts made of AISI 304 or 316L is equal to 97%.

## 1. Introduction

Austenitic stainless steels, such as AISI 304 and AISI 316L, have wide usage in various types of parts, mechanisms and constructions in many different industries. For instance, they have wide application in chemical and food industries, pharmaceuticals, medicine, and automotive, aircraft, and maritime industries, etc. The parts used in these industrial and life care fields are subjected to many strict requirements, such as high corrosion and wear resistance, surface hardness and mechanical strength, and must have high integrity contact surfaces with little roughness asperity, etc., in order to achieve the intended operational characteristics. Often, the operational conditions of their work include cyclic loads, which could lead to fatigue failures, resulting in significant shortening of their operational lifetime. While in some cases machine parts destroyed due to fatigue from mechanical devices are relatively easy and not too costly to replace, in other cases, such as orthopedic prostheses [1], this is not an easy and simple task. Therefore, the fatigue durability of such prostheses is of great importance for the patient. Destruction of mechanical parts due to fatigue or wear, for example, when food processing machines are used, can lead to small particles (debris) getting into the food, thus endangering the health and even the lives of consumers. Even an auxiliary part’s fatigue failure can cause severe operational issues, violation of the whole mechanism or even destruction of the entire device or machine.

There are many approaches developed over the years in order to minimize fatigue failures. They can be divided into two large areas: the first one is related to the design of the machine parts, and the second is related to the development of specific finishing processing technologies, which have led to improvement of fatigue strength.

Among the design approaches the most notable is “Infinite-life design” [2] which keeps all stresses below the limits of fatigue effects. This approach is used where safety and/or long life are more important than space and weight limitations. “Safe-life design” [3] allows fatigue cracks to occur during the operational period, but they never grow to a critical length. Some structures subjected to high stresses, such as aircraft wings and hulls, pressure vessels, and heavy duty bearings, employ this type of design. The designed “safe life” usually is about one-fourth of the predicted fatigue life. “Fail-safe design” allows cracks to occur, but they never lead to fatigue failure earlier than the scheduled maintenance. The maintenance process must detect, repair, or replace the damaged parts. This approach is employed mainly in the aircraft industry since the weight requirements are crucial and there are strict regulations for maintenance in comparison with other industries. “Damage-tolerant design” [4] includes fracture mechanics and takes into account the initial imperfections in the material structure. The approach is based on the assumption that imperfections (flaws, cracks, etc.) can exist in any structure, and these cracks propagate (and grow) during the usage period. This approach is commonly used in civil engineering, mechanical engineering, and aircraft engineering to manage the extension of cracks in structures by using the principles of fracture mechanics. In this approach, a corresponding specific maintenance program must provide detection and repair of accidental damage, corrosion, and fatigue failure before the structure residual strength is reduced below an allowable limit. Although the abovementioned fatigue-based design approaches have been implemented for a long time in engineering practice, it is still possible for fatigue failure situations to occur because of different shortcomings of design and omissions in implementation. There are many influencing factors which affect fatigue behavior [5], the most important of which are stress and strain concentrations; material properties and metallurgical factors; surface finish and directional properties; and type and nature of loading, etc.

Reduction of fatigue failures by using specific methods for parts production is the second strategic approach [6,7,8,9,10]. By choosing an appropriate processing method, the effect of various “defects”—caused by metallurgical factors, surface hardness, residual compressive or tensile stresses in the surface layer, surface roughness and topography, etc.—on fatigue endurance can also be significantly reduced. The surface roughness is of great importance among the technological factors because it is well known that fatigue failure usually originates on the surface of the part. There is evidence that, for steel, the higher the tensile strength, the more critical is the surface finish [11,12]. Because the surface condition has a significant effect on fatigue strength, some of the traditionally used finishing processes of machine parts, such as milling, turning, grinding, polishing, lapping, etc., cannot completely meet all requirements. The surface roughness after most of these processes has a typical topography with notches due to imperfections that occurred after removing the allowance. Residual tensile stresses and even cracks can occur after grinding, for example, due to intensive heating of the surface layer, which is usual for this finishing process [5,13,14].

A considerable increase of the fatigue strength can be achieved if finishing processes such as shot peening, deep rolling, ball burnishing (BB), etc., which are based on plastic deformation of the surface layer in a cold state, are applied [5,15,16]. After their application, compressive residual stresses are obtained in the surface layer, combined with the removal (or minimization of depth) of the notches from previous cutting operations [17]. Processes based on plastic deformation also increase the hardness of the surface layer, which favors wear resistance. When rolling or BB is applied to metastable austenitic stainless steels, such as the aforementioned AISI 304 and AISI 316L, the austenite (γ-phase) transforms into martensite (ε- and/or α’-) due to a recrystallization change caused by deformation energy, even at ambient temperature. On the other hand, a smooth surface topography with very little roughness (Ra criterion from 0.8 to 0.025 μm) can be achieved after rolling or BB is applied as a finishing process [18,19]. This way, two of the abovementioned negative effects of the cutting finishing methods on fatigue resistance can be avoided.

Ball burnishing has several different varieties, depending on the purposes for which it is applied [20]. They are all related to the plastic deformation of the surface layer, but can differ in the way the deforming element moves along the parts’ surface. When the goal is to achieve only surface smoothing, the classic BB schemes can be used [21], in which the trajectory of the deforming element is the same as that of the cutting tools in drilling, turning, and milling cutting operations. Recently, there has emerged more and more evidence that the BB process can be successfully implemented using computer numerical control (CNC) equipment, which allows application of different strategies and toolpaths [22,23,24,25]. Some authors [26] report improvements in BB tools that allow the simultaneous use of multiple deforming elements to increase the process productivity. Other authors [27] apply ultrasonic artificial vibrations to the deforming element in order to intensify the plastic deformation in the surface layer.

The BB process also can be applied in order to create a specific surface roughness (or so-called “regular reliefs”) in addition to improving the other aspects of surface layer integrity [28]. Regular reliefs (RR) are formed by the traces of plastic deformation that are left on the surface as result of the passage of the deforming element, which is pressed with a certain deforming force. Here the deforming tool must perform a more complex trajectory (in contrast to the classical one), involving additional oscillation with a certain frequency and amplitude. As a result, this type of BB process is called “vibratory ball burnishing” (VBB) [28]. The RR obtained by vibratory ball burnishing can be five different types, according to the classification given in [28], the most interesting of which is the IV-th type, which covers all of the processed surface and forms a completely new surface roughness. Depending on the regime parameters of the VBB, the RR of the IV-th type can have specific rectangular or hexagonal cell patterns, which significantly increase the ability to retain lubricants and micro debris and stop them from wearing the surfaces burnished by this method, in comparison with smoothed ones [29,30,31]. Although the roughness of the RR is greater than that of the smoothed surfaces, they offer better contact conditions, such as reaching liquid friction at lower speeds [32], optimizing the contact spots, and increasing the wear resistance, while the other advantages of the BB process remain the same.

When VBB is adapted for implementation by using a CNC lathe or milling machine, the complex toolpath for obtaining RR can be achieved by interpolation between the machine axes [33]. In this case, the additional reciprocating movement (or the “vibration” component) of the ball tool is not needed, which significantly simplifies its construction. The preliminary mathematical modeling [34,35] of the tool path and the higher precision of the CNC equipment give opportunity to obtain full or partial RR with higher dimensional precision and repeatability of the cells. In the present work this variant of the BB process was used in order to obtain RR of the IV-th type on flat surface of the test parts subjected to fatigue failure tests. This type of RR is expected to have no worse fatigue-failure behavior than the smoothed surfaces after applying other (traditional) variations of the BB process. The results from earlier conducted experiments with AISI 304 steel [36] support this expectation, but the obtained experimental data concern only few of the BB process conditions, and the statistical analysis used to evaluate the significance of the regime parameters of the process can be improved by using the Bayesian approach [37].

Based on the investigated sources, the current work’s main goal is to investigate the effect of RR of the IV-th type obtained by BB on the fatigue life of AISI 304 and AISI 316L austenitic steels. Secondly, it aims to study the effects of the main BB process regime and relief parameters and their interactions over fatigue life.

Finally, it aims to create a stochastic model for calculation of the probabilities of reaching a certain number of cycles until fatigue failure, for the possible combinations of “low” and “high” levels of the BB regime’s parameters for both investigated steels.

## 2. Materials and Methods

### 2.1. Materials

The materials used in this work were rolled sheets of austenitic stainless steels: AISI 304, provided by SARITAS Celik Sanayi ve Ticaret A.S. (Istanbul, Turkey) and AISI 316L, provided by Acerinox Europa SAU (Los Barrios, Spain), both with 4 mm thickness. The chemical compositions of these austenitic steels and their basic mechanical properties are given in Table 1.

### 2.2. Obtaining RR of the IV-th Type by BB Process, Implemented on CNC Milling Machine

#### 2.2.1. Calculating the Toolpath Trajectory of the Ball Tool

**Figure 1:**
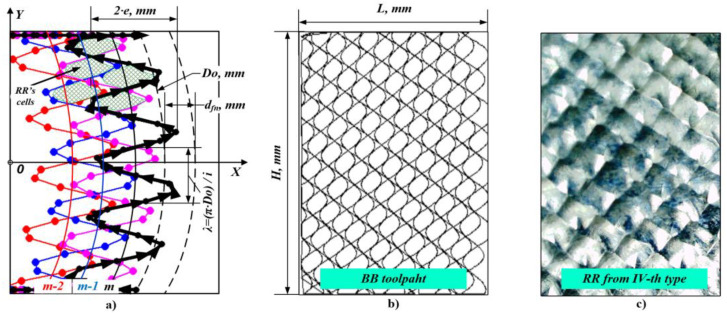
(**a**) Ball burnishing (BB) toolpath trajectory basic parameters; (**b**) BB toolpath distribution within the burnished surface boundaries; (**c**) resulting regular reliefs (RR) of the IV-th type with rectangular cells.

Because RR were formed onto planar surfaces in the present work, the needed kinematics for the BB process could be borrowed from the classical vibratory BB process [34], but adapted for implementation with a contemporary CNC milling machine. This way the needed complex toolpath of the deforming tool (shown in Figure 1a,b), which is essential for the formation of RR of the IV-th type (see Figure 1c), could be achieved much more efficiently, and with a greater accuracy.

If we take into account the principle of the CNC equipment programming (i.e., ISO code) that the tool moves from its current position to the coordinates of the next position according to the NC code, the complex toolpath needed in the BB process can be divided into a relatively large number of short rectilinear segments that interpolate it with a sufficient accuracy. The end point X and Y coordinates of each segment were calculated using the following system of functions [34] (see Figure 1a):

(1) $$|\begin{array}{c} X_{m,n}=\left(e\cdot \cos{\left(\frac{2\cdot \pi \cdot n}{p}\right)}_{m}+\frac{1}{2}\cdot \sqrt{D_{0}{}^{2}+4\cdot e^{2}\cdot \cos{\left(\frac{2\cdot \pi \cdot n}{p}\right)}_{m}{}^{2}}\right)\cdot \sin\left(\frac{2\cdot \pi \cdot n}{p}{+i}_{p}\cdot m\right)+d_{fn}\cdot m\\ Y_{m,n}=\left(e\cdot \cos{\left(\frac{2\cdot \pi \cdot n}{p}\right)}_{m}+\frac{1}{2}\cdot \sqrt{D_{0}{}^{2}+4\cdot e^{2}\cdot \cos{\left(\frac{2\cdot \pi \cdot n}{p}\right)}_{m}{}^{2}}\right)\cdot \cos\left(\frac{2\cdot \pi \cdot n}{p}{+i}_{p}\cdot m\right) \end{array}$$

where *p* is the number of the toolpath points; *n* is the index of the current point from the toolpath (*n* = 0, 1, 2,… *p*); *m* is the index of the current segment of the toolpath (*m* = 0, 1, 2,… *q*); $q=L/d_{fn}$ is the number of all toolpath segments; *D*_0_, mm is the toolpaths’ segment diameters; *e*, mm is half of the amplitude of the sinewaves; *d_fn_*, mm is the linear distance between the toolpath segments; *ip* is the fractional part of the ratio *i* = π·*D*_0_/*λ*.

The parameter *i_p_* determines the phase shift between sinewaves of the successive toolpath segments and can have values between 0 and 0.5. When *i_p_* ≈ 0.15, the RR have cells that resemble a hexagonal shape, and when *i_p_* ≈ 0.45 the cells are close to having a rectangular shape. The integer part of the parameter *i* sets the number of the sinewaves within each of the toolpath segments, thus determining their period *λ*, mm. It has an impact on the resulting RR cells’ size along the Y axis (see Figure 1a).

The parameters *e* and *d_fn_* from Equation (1) have a significant impact on the RR cells’ size along the X axis. One of the important requirements to be met is that *d_fn_* must be equal or less than *e* (i.e., *d_fn_* ≤ *e*) in order to guarantee obtaining RR of the IV-th type. Otherwise, if *d_fn_* > *e* there is a possibility of obtaining RR of types I-st, II-nd or III-th, which can be formed onto burnished surfaces, which contain “isles” with initial roughness obtained by the previous operation. This is undesirable because it can lead to non-uniformity of the physical and mechanical properties in the burnished surface layer. When the values of these parameters are set in the Equation (1), the results for the imprint diameter also must be taken into account.

Another important condition is that the toolpath points must be generated only within the burnished area boundaries, because there is no reason for the deforming tool to process the space outside the material. In [34] an algorithm is presented which is based on additional logical conditions to prevent generation of points outside the material boundaries. It also connects the sinewave segments with each other (see Figure 1a) and this way ensures the overall length of the toolpath is as short as possible. The outcome of the algorithm is a single polyline, defined by the points calculated by Equation (1), with an optimal length that depends on the shape and size of the area processed by the BB operation. The polyline created in this way can then be exported as a two-dimensional drawing (in DXF or DWG format), and be used in suitable CAM for further modeling of the BB operation.

#### 2.2.2. Preparing the NC Code for the BB Operation

Unfortunately, the numerical control (NC) code cannot be written by hand in this case, because of the great number of points (sometimes several hundred thousand points) that the BB operation toolpath usually contains. The numerical control system of the most widespread milling machines also do not have preinstalled appropriate canned cycles that can be used for that purpose. However, the NC code needed to perform the BB operation in a suitable CNC milling machine can be obtained automatically, using appropriate CAM software. What particular CAM software is used is not of a great importance as long as it has a suitable milling component that allows the tool to be guided along a predetermined curve (in the present case the polyline, generated in the previous step), and a suitable postprocessor for the specific CNC milling machine that will be used.

After postprocessing the modeled toolpaths in CAM for all the BB operations according to the experimental designs, the corresponding NC codes were obtained.

### 2.3. Fatigue Failure Test Setup

#### 2.3.1. Description of the Experimental Method and Setup

**Figure 2:**
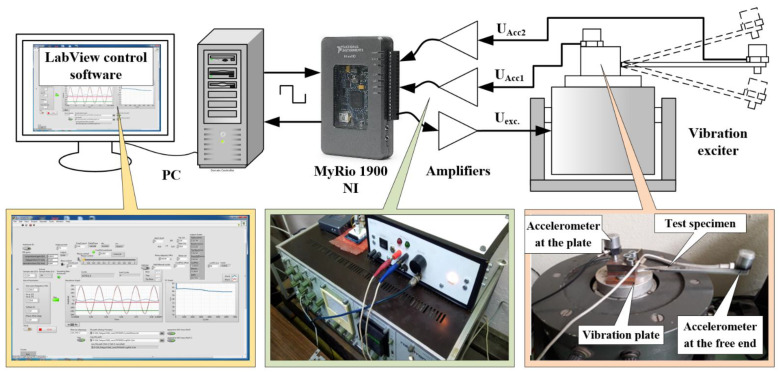
Components of the fatigue failure experimental setup.

A reversal bending fatigue test similar to that in [36] was performed on a vibration exciter (see Figure 2). The test specimen was mounted as a cantilever beam on the exciter’s vibration table. Two accelerometers were mounted: one at the free end of the specimen and the other at the exciter table. The vibration exciter was harmonically excited with a frequency close to the fundamental resonance mode shape of the specimen.

As the difference between the tip and base acceleration is proportional to the applied stress, the load was controlled by adjusting the amplitude and the frequency of the shaker signal during testing. An algorithm similar to those realized in [38] was developed and installed in the MyRio 1900 (National Instruments, Austin, TX, USA) for automatic adjustment of the excitation signal frequency in order to keep the applied stress constant during the test. The test was stopped automatically when the algorithm was unable to maintain the preset stress amplitude by adjusting the frequency of the exciting signal. In order to ensure the stable working of the self-adjustment algorithm, the frequency of the exciting signal must be a little greater than that at the resonance. This way, the possibility of the exciting signal frequency starting the amendment in the wrong direction is eliminated. Using a personal computer and the LabView (National Instruments, Austin, TX, USA) visual programing environment, software was developed to monitor the experimental parameters and to count the cycles until fatigue failure of the specimens. The software saved incoming data from MyRio 1900 as logs, which were used for further analyses of the results.

All the specimens in the current experimental investigation were tested at equal loading, in order to investigate the impact of the BB regime parameters on fatigue failure.

#### 2.3.2. Determining Experimental Conditions of the Fatigue Failure Test Using FE Analysis

**Figure 3:**
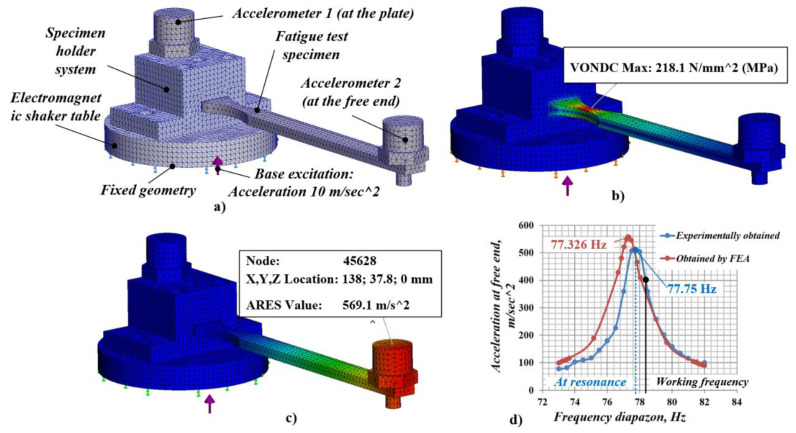
(**a**) Meshed 3D model with fixed geometry and excitation applied with (**b**,**c**) results from finite element analysis (FEA) for maximal obtained stress and acceleration at the free end of the specimen and (**d**) response graphs of “frequency-acceleration” (amplitude) obtained after FEA and experimental study conducted.

The purpose of the conducted finite element (FE) analysis (see Figure 3a–d) was to determine the vibration fatigue test setup parameters, in order to assure invariable conditions of the fatigue failure experimental investigation. A linear dynamic study was carried out for the investigated materials. It was based on natural frequencies and mode shapes to evaluate the response of the test specimens to dynamic loading, caused by shaker’s table.

To conduct the FE analysis study, a SOLIDWORKS Simulation module (Education edition 2020–2021, SolidWorks Corporation, Waltham, MA, USA) was used. The input parameters in the FE model included the test specimens’ material properties (see Table 1) and applied uniform excitation (acceleration) of 10 m/s^2^ which was restrained only in the vertical direction (see Figure 3a). The masses of the test specimens and the used acceleration sensor at the cantilever’s free end were set in the FE model, based on weight measurements of the real items, using a precise scale. The global damping ratio for each mode was set to be 0.01.

As the 3D components participating in the assembly had relatively simple geometric shapes, a standard high-quality mesh was used with the global size of the elements being 2.0 mm, and tolerance 0.1 mm. As a result, the total number of the obtained mesh elements was 66,382, and they had 99,510 nodes.

The obtained FE model results for maximum (Von Misses) stress (218.1 MPa) at the concentrator of the fatigue specimen, along with displacement (2.36 mm), and acceleration (569.1 m/s^2^) at its free end (shown in Figure 3b–d), were calculated for the resonant frequency (77.326 Hz). The last two parameters were determined in the mesh model with the number 45,628, which was located as near as possible to the center of gravity of the accelerometer (see Figure 3c) in order to bring the simulation analysis as close as possible to the actual experimental conditions.

Using real test specimens (without RR formed by ball burnishing) and adjusting the electromagnetic shaker to sweep the frequency diapason around the obtained resonance frequency, a “frequency-acceleration” (amplitude) response graph was obtained. A similar response graph also was obtained from the FE model. Both graphs are shown together in Figure 3d for illustration. The comparison between them shows that the FE model gave results close to those obtained after the physical test. The resonant frequencies of the measured (77.75 Hz) and the calculated (77.33 Hz) response graphs differ by only 0.5%. The difference in respect to the maximum acceleration values at resonance between the measured (531 m/s^2^) and the calculated (557.7 m/s^2^) response graphs did not exceed 5%. Therefore, the results derived from the FE model can be considered as adequate.

Due to the algorithm implemented to automatically adjust the frequency of the excitation load of the exciter, which was used to control the acceleration at the free end of specimen and thus to keep the stresses obtained in the concentrator constant during the fatigue test, it was not advisable for the exact resonant frequency (i.e., the peak of the response graphs) to be chosen as a work point. In order to guarantee stable working of the auto-adjusting algorithm, the work frequency of the shaker had to exceed the resonant one. Using the FE model, and the obtained response graph, it was possible to specify previously the suitable initial working frequency and the corresponding acceleration at the free end, which defined the resulting stress at the concentrator of the fatigue specimens. This way the FE model facilitated the preparation of the fatigue failure experimental investigation, and allowed us to reach optimal experimental conditions without the need of carrying out many preliminary physical tests.

## 3. Experimental Research

In order to investigate the impact of the main regime parameters of the BB process on fatigue failure effects, an experimental investigation was conducted according to the first goal. Its purpose was to reveal the main effects and interactions between the BB regime parameters: deforming force—*F*, N and feed rate—*f*, mm/min, which were expected to a have major influence on the degree of the plastic deformation in the surface layer of the material, and the toolpath trajectory parameters: *e*, mm and *i*, which are known to determine the shape and the size of the RR cells. The experimental research was based on the approach of the full factorial designs [39]. The selected four factors (i.e., the BB regime parameters *F*, *f*, *i*, and *e*) varied on two levels—“low” and “high”. The experimental design executed is shown in Table 2.

As a response criterion, the number of cycles until fatigue failure was used. For every trial, included in the experimental design, two replicates were performed. The obtained results were subjected to several different analysis techniques, including the Pareto, *t*-test, and Bayesian rule.

The natural values of *i*, and *e* were selected on the basis of the previous research of the authors [40]. Their low and high levels were set to obtain RR with different size cells. In Table 3, the four toolpath types which had different unfolded lengths, derived by using Equation (1), are illustrated. The unfolded toolpath length gives the relief degree of imbrication, which is proportional to the number of passes in conventional burnishing technology.

The values for *F* and *f* were selected from the operational capabilities of the ball burnishing tool [41], and those of the CNC milling machine (3-axes HAAS TM-1, USA [42]) used.

The rest of the BB parameters, which were not varied during the experiments, were fixed as follows: diameter of the deforming element was *dc* = 14, mm; number of points of the toolpath *p* = 10,000; the distance between toolpath segments *d_fn_* was set to be equal of half of the sinewave amplitude *e*. In order to avoid jamming of the ball tool, Mobil DTE 25 was used as a lubricant for all ball burnished experimental specimens.

**Figure 4:**
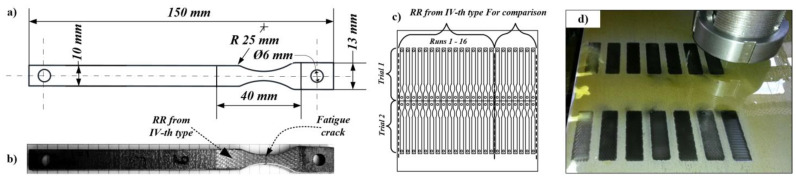
(**a**) Fatigue test specimens’ shape and dimensions; (**b**) BB area with RR of the IV-th type; (**c**) arrangement of the specimens within the plates of AISI 304 and AISI 316L. (**d**) Physical BB-operation for obtaining the real RR from the IV-th type.

The test specimens were made of austenitic stainless steels AISI 304 and AISI 316L, described in Section 2.1. They had a specific shape and dimensions, shown in Figure 4a,b. The specimens had two stress concentrators whose purpose was to guarantee the fatigue cracks developed in the narrowest section of the material (see Figure 4b), i.e., where the RR were formed after applying the BB operation. According to the experimental design shown in Table 2, 16 × 2 = 32 specimens were processed by BB for AISI 304 steel and another 32 specimens with the same characteristics for AISI 316L steel, respectively. Fourteen additional specimens (without RR) were also made of both of the steels (see Figure 4c,d). Their purpose was to adjust the fatigue test setup, as well as to compare the results obtained for the number of cycles to fatigue failure with and without the application of the BB operation.

The BB operation was applied on both sides of the plates, with one and the same combinations of the regime parameter values, according to experimental design, shown in Table 2. Thus, each specimen processed by BB had two-sided RR. After all RR were formed onto both sides of the plate, the different specimens were cut off the plate using a CO_2_ laser cutting machine MAZAK SUPER X48-Champion. In order to avoid the impact of the sharp edges after the laser cutting operation at the stress concentrator on fatigue life of the specimens, they were subjected to electropolishing.

## 4. Results

### 4.1. Preprocessing Data

As a result of the experiments, the fatigue testing number of cycles to failure (*Nf*) was obtained. According to the experimental plan, specimens of 304 and 316L steels were burnished with 16 combinations. Low and high levels of the factors were coded with −1 and 1. Levels of two- and three-factor interactions were calculated as a multiplication of main factor levels.

The specimens of both steel batches were tested successively at each combination (replication r = 4), resulting in a total of 16 × 4 = 64 specimens (32 specimens of each kind of steel). The descriptive statistics of the fatigue data are shown in Table 4. Considering that unlike AISI 316L, specimens of AISI 304 were notched, the fatigue life results were as expected. The 316L specimens’ fatigue life lay predominantly in the 10^6^ range, while the fatigue life of 304 specimens lay predominantly in the 10^5^ range.

The fatigue life, even at constant stress amplitude, showed stochastic behavior. The materials’ fatigue resistance due to the randomness of microdefect distribution, loading condition variations, and specimen preparation were the main sources of uncertainty. To model fatigue life, Normal and LogNormal distributions are commonly used.

Of particular interest in this study was the increase of the fatigue life due to burnishing operation with different combinations of regime parameters. To identify the characteristics of fatigue life gain, additional experiments with non-burnished specimens were carried out at the same loading conditions. The mean result for 304 steel was 2 × 10^4^ cycles (tested four specimens). For 316L steel, the mean result was 14 × 10^4^ cycles (tested three specimens). Based on these results, value *logCycles* representing gain of the fatigue life due to burnishing were formed. Cycles to failure of burnished specimens are divided into base cycles of non-burnished specimens and ones, converted to logarithmic scale, using the decibel rule to get more physical meaning, (Equation (2))

(2) $$logCycles=20\cdot log\left(\frac{cycles\;to\;failure\;of\;burnished\;specimen}{cycles\;to\;failure\;of\;base\;specimen}\right)$$

Descriptive statistics of data converted to log scale are shown in Table 4. These data are considered as primary data for the statistical analysis.

**Figure 5:**
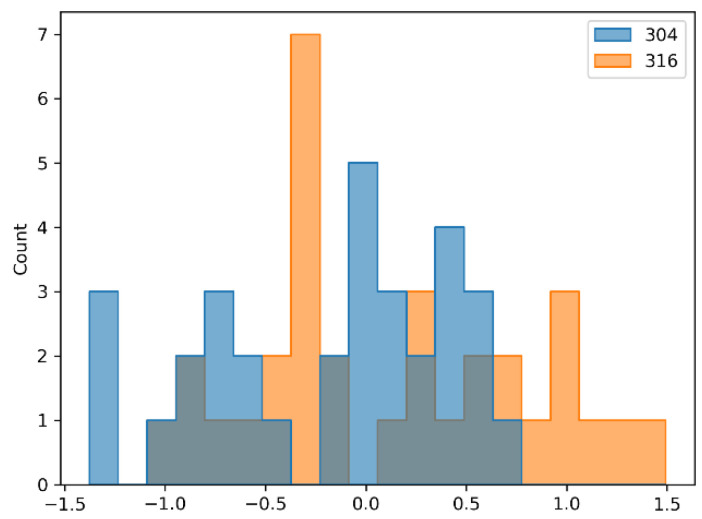
Scaled data histogram.

To ensure comparability of the data for different materials, the logCycles were scaled using a robust scaler from the Python library “SciKit Learn” [43]. This type of scaler uses the first and third percentile values and is more robust to outliers Equation (3). This kind of scaling can be applied to additional data (if replication of the experiment is made). The histograms of scaled data are shown in Figure 5.

(3) $$y_{scaled}=\frac{y_{i}-q_{1}\left(0.25\right)}{q_{3}\left(0.75\right)-q_{1}\left(0.25\right)}$$

Most of the data were in the unit range (−1; +1). The whole range of data was (−1.5; +1.5). As shown in Table 5, for the particular materials included in this research, mean and *q*_1_(*0.25*) and *q*_3_(*0.75*) quantiles were quite similar and scaling just centered the data and converted standard deviation to 1.

### 4.2. Effects and T-Test

**Figure 6:**
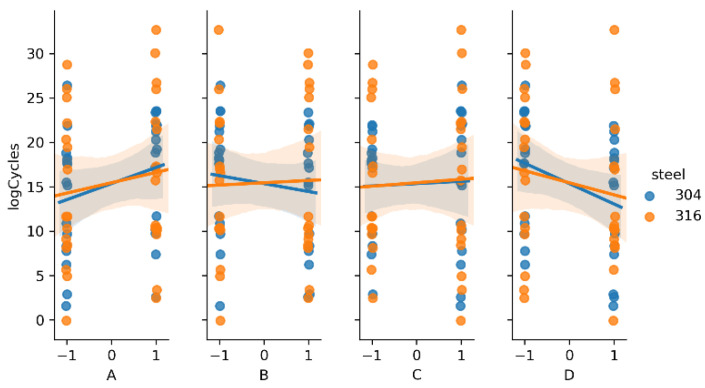
Regression plots representing the main effects.

**Figure 7:**
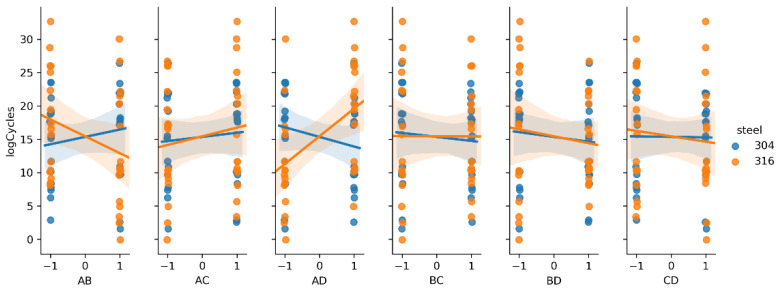
Regression plots representing two-factor interactions.

**Figure 8:**
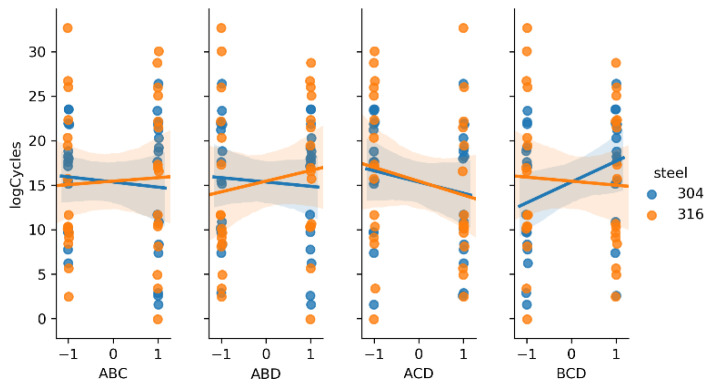
Regression plots representing three-factor interactions.

The main effect of a given factor is the mean difference in the level of response as the input moves from the low to the high level [44]. Combined two- and three-factor effects (interactions) are calculated by multiplication of the main factor levels. For example, two-factor interaction is positive when the inputs move in the same directions and negative when the inputs move in opposite directions. To visualize these effects, linear regression plots are presented on Figure 6, Figure 7 and Figure 8. Each kind of steel is treated separately for comparison.

In previous research [36], only the results of fatigue testing of AISI 304 steel were analyzed. It was found that the main factors A and D, and the interactions AC and AD, had the greatest impact. The new data from the 316L steel fatigue testing showed similar behavior, according to the main factors. In contrast, interaction AD was very strong, but the slope was different, comparing steel types (positive for 316L and negative for 304), as shown in Figure 7. From the three-factor interactions, ACD seems to be important (Figure 8).

**Figure 9:**
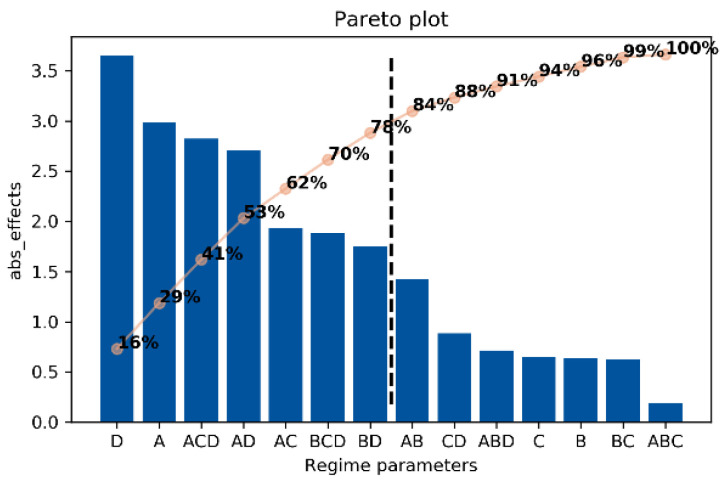
Pareto plot factors (regime parameters) and interactions.

Since steels were burnished within the same experimental plan, and the fatigue testing procedure was the same, similar effects should be expected, so the new effects calculation was made considering the whole dataset. The main effects and interactions are given in descending order of their absolute values in Table 6. A Pareto chart (see Figure 9) shows that the first seven factors from the table should be considered significant (D, A, ACD, AD, AC, BCD, BD), because they covered about 80% of the total effect.

The obtained results were confirmed by *t*-test. This test checks the null hypothesis if the mean values of the two groups of samples are identical. The *p*-values, calculated from the *t*-test, are given in the last column of Table 2. All of them were greater than 5% (*p* > 0.05), which means that the null hypothesis cannot be rejected with 95% level of confidence for all the factors and interactions. If value *p* = 0.1 (90% confidence) is taken as a significance level, the null hypothesis can be rejected for D and A factors and these factors are considered significant for the regression model. The values of these factors confirm the general findings for the burnishing processes: that higher force (A) and low federate (D) guarantee a higher degree of plastic deformation, thus benefiting the fatigue life [28], but give no information about the influence of the regular relief degree of imbrication, connected with factors B and C.

More detailed statistical inference from such noisy data can be obtained, using the Bayesian approach. Bayesian models are called probability models, since as a result distribution rather than the point estimates for the unknown parameters are obtained. According to the Bayesian rule Equation (4), the posterior probability of parameters of interest θ, based on the observed data *y*, can be estimated, using our prior knowledge about these parameters (θ). The term P(y|θ), called likelihood, is a probabilistic model for the data. The denominator term P(y) is the marginal probability of the data, called evidence. Since it is just a normalizing constant, it can be omitted and stated that posterior probability is proportional to likelihood times, prior probability.

(4) $$P\left(\theta |y\right)=\frac{P\left(y|\theta \right)\cdot P\left(\theta \right)}{P\left(y\right)},$$

where P(θ|y)—posterior; P(θ)—prior; P(y|θ)—likelihood;

$P\left(y\right)=\int^{\;}P\left(y|\theta \right)\cdot P\left(\theta \right)d\theta$—evidence.

P(θ|y) ∝ P(y|θ)·P(θ)

For the continuous random variables, *P*(*θ*|*y*) is a probability density function (PDF) of a certain distribution. So, Bayesian modeling requires first setting the appropriate likelihood distribution, which describes how the data can be generated, and second, choosing the prior probability distributions for all the unknown θ parameters. Prior distributions can be constructed as non-informative, i.e., diffuse or even improper, which PDF does not integrate to 1. If too informative (strong) prior distributions are used, there is a risk in ignoring the experimental data. Of course, in the presence of enough data, the prior choice is ignored, and likelihood dominates the posterior distribution. The Bayesian probabilistic model can be updated if new data are available just by setting the posterior distributions to prior for the new data.

For some of the simple cases, a closed form solution for the posterior density is given in the literature [37]. Nevertheless, for all probabilistic models, Bayesian inferences can be made just by simulation. To ensure random, non-correlated samples covering the whole distribution, Markov chain Monte Carlo (MCMC) sampling algorithms are used. Nowadays, numerous statistical packages and libraries for “R” and Python are available. Some of the most popular are WinBUGs, JAGS, Stan, and PyMC.

### 4.3. Regression Model

#### 4.3.1. Ordinary Least Square Regression (OLS).

Ordinary least square linear regression Equation (5) assumes Gaussian distribution of the noise.

(5) y=Xβ+ϵ,

where $y\;\in \;{\mathbb{R}}^{n\times 1}$—column vector of parameter of interest (regressors); $X\;\in {\mathbb{R}}^{n\times k+1}=[\left\{1\right\},\;\{x_{1}\},\;\left\{x_{2}\right\}\ldots \left\{x_{k}\right\}]$, predictors (or design matrix); $\beta \in \;{\mathbb{R}}^{k+1\times 1}$—column vector of regression coefficients; $\epsilon \sim \;N\left(0,\;\sigma^{2}I\right)$—Gaussian noise.

As the effects and interactions are calculated, a linear regression model can be formed as Equation (6), where the first term is a mean of the dependent variable *y*, and to calculate the other coefficients *i*^th^ effect or interaction value should be divided by 2, since it shows the amount of change of the regressor as predictor *x_i_* moves 2 steps, from −1 to +1.

(6) $$\hat{y}=X\beta =\;\bar{y}+\frac{1}{2}\overset{k}{\underset{i=1}{\sum }}\sum^{\;}effect_{i}\times x_{i}$$

The probabilistic model of linear regression can be expressed as Equation (7). Data *y_i_* comes from Normal distribution with a mean equal to OLS estimation $\hat{y}$ and variation $\sigma^{2}=\epsilon$.

(7) $$y_{i}\;∽N\left(X\beta ,\;\sigma^{2}\right),$$

where *i* = 1,2,…,*n* is the number of data points

From a Bayesian perspective, each regression coefficient can be treated as a random variable, coming from a Normal distribution with unknown mean and variance Equation (8).

(8) $$\beta_{i}\;\sim \;N\left(\mu_{i},\;s_{i}^{2}\right),$$

Under a non-informative Jeffrey’s prior, this problem has a closed form solution [37]. The resulting parameters of posterior are dominated by experimental data. The marginalized distribution for mean *μ*, with the integrated *s*^2^ is a non-centered Student-t distribution Equation (9).

(9) $$\mu_{i}\;|\;y\;\sim \;t_{n-k-1}\;\left(m_{i},\;S_{i}^{2}\right)$$

The location parameter m_i_ is equal to the least squares estimation of regression coefficients, the scale parameter $S_{i}^{2}$ is equal to the standard error, and the degrees of freedom are equal to the degrees of freedom of the regression model. From this distribution, credible intervals CI and/or high-density intervals (HDI) with a certain probability can be formed.

Results from the OLS regression, including the first seven factors from the Pareto plot, are given in Table 7. To perform this regression, the Python library Statsmodels was used [45].

Looking at the 95% HDI, again the conclusion is that there was no significant factor with a significance level α = 5%.

#### 4.3.2. Robust Regression

**Figure 10:**
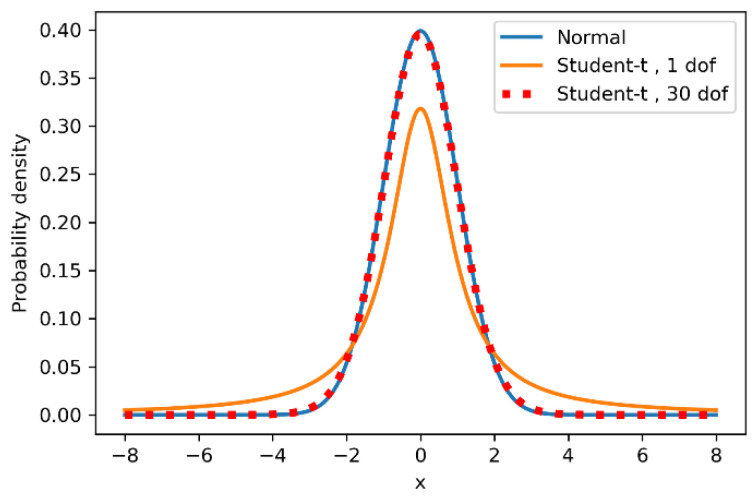
Student-t and normal distributions.

To decrease the influence of the outliers, a new probabilistic model was built. The response variable were modeled with a Student-t distribution. This distribution has the 3rd parameter ν (degrees of freedom), which controls the tails [46]. Lower values of ν lead to the distribution high tails and the regression is more robust to outliers. At about ν >30, the Student-t distribution coincides with the normal (Figure 10).

The parameters of the distribution were random variables, for which the appropriate prior distributions should be given. For regression coefficients, non-informative (Normal with high variance) were chosen. The degrees of freedom can be fixed or if treated as a random, variable exponential prior can be given.

(10) $$y\;\sim \;t\;\left(\;\hat{y},\sigma^{2}\;,\;v\;\right),\;\sigma^{2}\sim \;HalfCauchy\left(\;s_{0}\right)\;v\sim Exp\left({\;\lambda }_{0}\right)\;\beta_{i}\;\sim N\;\left(\mu_{0}{}_{i},\;\sigma_{0}{}_{i}^{2}\right)$$

where v degrees of freedom; *s*_0_, *λ*_0_, *μ*_0_, *σ*_0_—known parameters.

For sampling this Bayesian model, the PyMC3 Python library was used [47]. This probabilistic programming language uses clear and easy to understand syntax. In particular, the PyMC3 generalized linear model module (GLM) is used to define this model. It, by default, sets the non-informative priors for the regressors $\beta_{i}\;\sim N\left(0,\;10^{6}\right)$. The model was defined just in 1 row with a string for the regression formula, data, and the family parameter, which set the likelihood to Student-t distribution (default was Normal). The degrees of freedom were fixed to ν=1. To sample from the mode, two independent Markov chains with length of 6000 samples were generated. The first 1000 samples of each chain were “burned” to avoid correlated samples. By default, PyMC3 uses the gradient based No-U-Turn-Sampler (NUTS).

**Figure 11:**
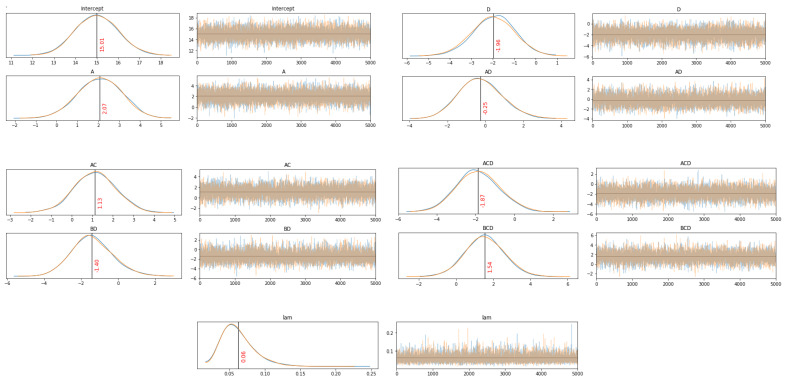
Trace plots (left—distributions; right—chains)

After sampling the trace, a plot of posterior distributions was available. On the right, the generated Markov chains were presented, and on the left, the posterior distribution density of the model parameters. As can be seen from Figure 11 they converged well. There were no abrupt changes, patterns, or other weird observations.

More detailed inferences about sampling can be made by reviewing the summary statistics (Table 8). First, the r-hat for all parameters was 1.00, indicating that simulated chains came from one distribution. Effective sample size (ess_mean) shows the number of non-correlated samples in the chains. They should be more than 10% of draws. It is seen that for all the parameters, the values were in the range of 5000–7000 from 10,000 draws.

**Figure 12:**
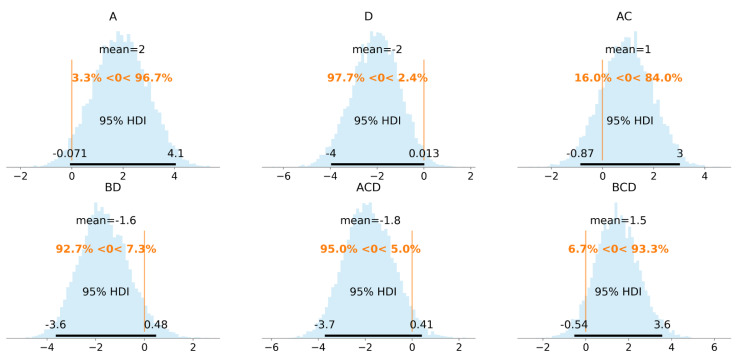
Posterior distributions of the proposed model coefficients. Histogram plot with 95% HDI and null reference value.

Intercept’s posterior mode was very close to the data mean (15.4). Posteriors of the regression coefficients were significantly away from zero, with the exception of AD coefficient posterior. It was centered close to zero, which indicates that it should be excluded from the model, unlike the previous OLS model, where its mean was positive with 95% HDI, predominantly on the positive side. To inspect other coefficients’ posteriors in detail, histogram plots with a highlighted 95% HDI and vertical line at zero as a reference value were plotted (Figure 12).

The posterior distributions of the regression coefficients AC and BD were not clearly on one of the sides (16% and 7.3% of the posterior lay below or above the reference value, respectively). Since these two factor interactions (AC, BD) were correlated with the three-factor interactions ACD and BCD, they can be considered excluded from the model due to overfitting.

The proposed probabilistic model can be further improved by centering the data by using the scaled data, setting the weakly informative priors to regression coefficients and the degree of freedom. The weakly informative is a prior distribution that covers the data behavior and generates the data at a reasonable scale but is not so strong as to influence the posterior. In addition, statements such as “weakly informative” depend crucially on what questions are being asked [48].

$$y_{centered}\;\sim \;t\;\left(\;X\beta ,\sigma^{2}\;,\;v\;\right)$$

(11) $$\sigma^{2}\sim \;HalfCauchy\left(\;s_{0}\right)\;v\sim Gamma\left(2,\;0.1\right)+1\;\beta_{0}\;\sim N\;\left(0,1^{2}\right)\;\beta_{i}\;\sim N\;\left(0,1^{2}\right),\;\mathrm{for}\;i=1,2\ldots k$$

**Figure 13:**
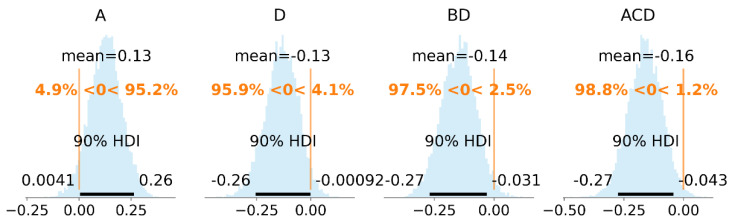
Posterior distributions of the final model coefficients. Histogram plot with 90% HDI and null reference value in scaled units.

Following some of the recommendations in [49], the Normal distributions with variance 1^2^ for the regression coefficients and Gamma (2, 0.1) prior for the degrees of freedom were chosen Equation (11). By trying different numbers of regression coefficients, the final model consisted of four regression coefficients A, D, BD, ACD. The posterior histograms with a 90% HDI are given in Figure 13.

## 5. Discussion

**Figure 14:**
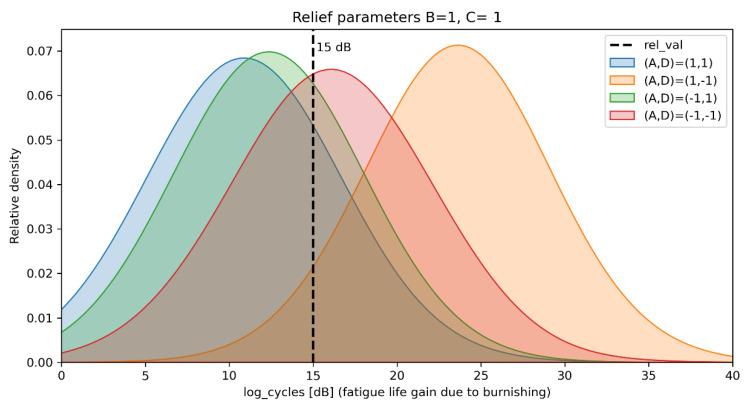
Posterior distributions of the fatigue life gain for relief with high degree of imbrication.

The proposed probabilistic model can be used for decision making. For example, in a lot of applications, a relief with certain parameters is needed, i.e., length and amplitude of the sinewave (B and C factors) are chosen due to technological requirements. So, the probabilistic question could look like: “Which force-feed rate (A–D) combination will give higher probability of reaching fatigue life gain of more than mean value (15 dB)?” To answer the question, the posterior distribution for the data (*y*) is formed by reversing the scaled data, fixing the regressors at a certain point, and incorporating the uncertainties of regression coefficients and data. For a relief with finer sells or a high degree of imbrication (B = 1, C = 1) the posteriors are shown in Figure 14. The probabilities *p*(*y* > *15*) calculated from the posteriors, for all combinations of relief parameters, are given in Table 9.

As an amplitude of the sinewave (factor C) gives higher effect to the relief’s degree of imbrication (see Table 9), for reliefs with high C value the feed rate should be kept low. For these kinds of reliefs, the optimal burnishing parameters are high force and low feed rate (A, D = 1, −1). For reliefs with low C value, the optimal burnishing regime is strongly dependent on sinewave length (factor B). If relief with a high B is needed, the feed rate (factor D) should be kept to a low value, unlike the relief with low B value, where high force and feed rate (A, D = 1, 1) are needed. Of course, all kinds of relief, using A, D = 1, −1 combination, result in the probability of reaching more than 15dB fatigue life gain, more than 50%.

The results from the proposed model support in general the published experimental results for conventional ball burnishing. Travieso-Rodriuez et al. investigated the fatigue life of burnished carbon steel specimens [50]. Their experimental results show that increasing the burnishing force and number of passes benefits fatigue life of the specimens. Rich experimental data for the influence of the burnishing regime on the fatigue strength is presented by Swirad [6]. He used a diamond composite burnishing element on low-alloyed carbon steel 40HM. The results showed that by increasing the burnishing force fatigue, the strength increased only to a certain threshold value. Beyond the threshold value, the fatigue strength decreased rapidly. Maximov et al. reported similar results for the influence of the burnishing force [51]. They used a diamond tool on aluminum alloy specimens and registered decrease of the fatigue life for the higher values of the burnishing force.

Other results of Swirad relate to the influence of feed rate and velocity. For burnishing tools with higher diameters, increasing the feed rate slightly decreases the fatigue strength after a certain value and the change of the velocity seems irrelevant.

The microstructural analysis of the burnished AISI 304 specimens given in [52] reports the phase composition in the surface layer. After the fatigue testing, strain-induced martensite is developed. In specimens with a large content of the martensite phase, a shortened fatigue life has been registered. Since for austenitic steels the martensite phase is strain-induced, the higher martensite content in some of the specimens can be a result of the local fluctuation of the material properties or the burnishing force (burnishing is done on rolled sheets without annealing). In [53] the effect of strain-induced martensite on fatigue behavior is investigated. Martensitic transformation is registered during the fatigue tests. The more pronounced transformation is for prestrained specimens. In [54], a strong influence of martensitic content on fatigue limit is emphasized and optimum martensite content for a predeformed specimen of 26% is reported. These phenomena look like a reason to adopt a two-side posterior distribution (p (ACcoeff < 0) = 16%) of AC regression coefficient in the first robust regression model (see Figure 3). Obviously, the chosen high value for burnishing force, combined with a high value of relief amplitude, is just below the threshold level and small random fluctuations can shorten the fatigue life.

In the above cited reference [52], a comparison of microhardness profiles of two burnished specimens is given, and the only difference in the relief is the sinewave length (factor B). There is no difference in the hardened layer depth. The only difference is the higher microhardness value, registered just below the surface in the specimen, burnished with a high B value regime, resulting in a slightly higher fatigue life. This phenomenon is captured by BD regression coefficient, whose posterior distribution lies on the negative side. This means that a low feed rate (factor D) in combination with a high sinewave length value (factor B) gives an additional improvement to the fatigue life.

## 6. Conclusions

The results from the conducted experiment show that the performed BB process on the test specimens leads in general to increase of the number of cycles until fatigue failure for both steels investigated. The gain in the fatigue life is more than 10 dB (about three times) for 75% of the AISI 304 and AISI 316L specimens, in comparison with those which had only preliminary plastic deformation, obtained after the steel sheets were rolled by the manufacturer (i.e., those without formed RR after applying BB). Thus, the formatted RR, after presenting a modification of the BB process, do not affect negatively the fatigue life results for these two steels. This is because the ridges of the RR cells’ boundaries do not play the role of stress concentrators which cause the formation of microcracks. This can be reported as an important operational characteristic for those parts which have RR, formed by using BB on their contact surface, in order to ensure a low slip resistance and a low wear, due to increased abilities to retain lubricants, dust, and/or debris which causes wear, in comparison with the smooth surface topographies, obtained after other traditional finishing processes, such as grinding, polishing, traditional ball burnishing, etc. Surfaces with RR could be part of equipment which works in highly dusty, abrasive or saltwater environments in marine, mining, petroleum, or chemistry industries, etc., and for which there are also requirements for high fatigue strength. The experimentally obtained results give us grounds to recommend this variant of the BB process, in which a specific RR of the IV-th type could be formed as a suitable finishing operation for such parts, subjected to both cyclic loads, for work in high-wear operating conditions. Using the advances of the contemporary CNC production equipment, and the presented approach for mathematical modeling of the toolpath of the ball tool, allows BB to be carried out as a finishing operation on the same machine, along with the previous cutting operations. This makes the BB operation easy to add to standard (generic) sequences of manufacturing operations for the production of such machine parts.

As can be seen from Table 9 and Figure 14, the optimal combination of the BB regime’s parameter values, in order to maximize the probability (up to 97%) of obtaining the maximum fatigue life of the parts made of AISI 304 or 316L, is A = 1, B = 1, C = 1 D = −1. In other words, the deforming force *F*, *N*, the amplitude of the sinewaves *e*, *mm*, and their number *i* must be set at their high values. However, the parameter feed rate *f*, mm/min must be set at its low values.

The presented approach for using the factorial experiment designs and Bayesian rule for data analysis reveals some tendencies about the impact of the main regime parameters of the BB process and their iterations on the fatigue life of the investigated steels. It provides good enough results in case of experimental investigations, in which it is not appropriate to perform a large number of trials, and the obtained results for the investigated parameter (i.e., fatigue failure cycles in our case) can have comparatively high variance. This can significantly shorten the time and facilitate the efforts for obtaining the needed results, in order to determine the optimal combination of BB regime parameters values in manufacturing conditions.

The methodological sequence for fatigue failure testing presented in the current work can also be applied to other materials, processing methods, and experimental plans, involving a different number of influencing factors. Our future work will be focused on its development and improvement in future research, similar to that presented in this paper.

## Figures and Tables

**Table 1:** Chemical compositions and mechanical properties of austenitic stainless steels AISI 304 and AISI 316L.

Chemical Compositions %										
Material	C	Cr	Mn	Mo	N	Ni	P	S	Si	Co
AISI 304	0.021	18.20	1.550	**-**	0.059	8.100	0.031	0.001	0.380	-
AISI 316L	0.022	16.63	1.285	2.031	0.050	10.065	0.030	0.004	0.340	0.226
**Mechanical Properties**										
**Material**	**Yield** **Strength, MPa**		**Tensile** **Strength, MPa**			**Elongation** **A5, %**		**HRB**		
AISI 304	324.00		626.00			55.00		188.0		
AISI 316L	353.15		628.58			49.18		82.0		

**Table 2:** Levels of factors (i.e., regime parameters of the BB operation).

Factor	Code	Low Level (−1)	High Level (+1)
Deforming force, *F*, N	A	1060	1735
Number of sinewave wavelengths, *i*	B	600.15	1200.15
Amplitude, *e*, mm	C	1.0	2.5
Feed rate, *f*, mm/min	D	150	300

**Table 3:** Unfolded length of the toolpaths at chosen factor level combinations.

Factor Level	Low Number: B = −1	High Number: B = +1
Low amplitude: C= −1	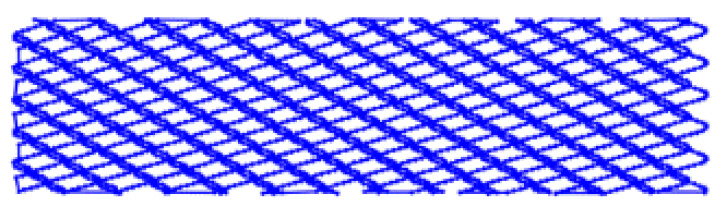 Toolpath length: 1011.75 mm	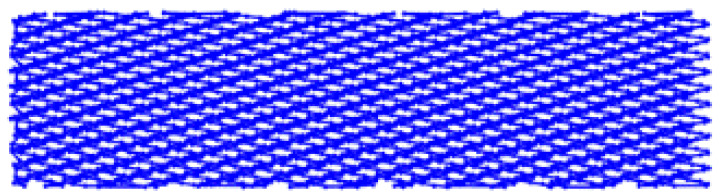 Toolpath length: 1783.25 mm
High amplitude: C= +1	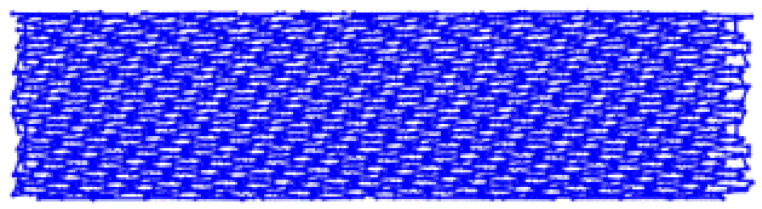 Toolpath length: 2231.34 mm	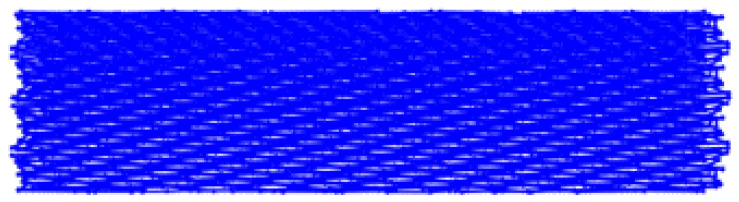 Toolpath length: 4164.44 mm

**Table 4:** Results from the fatigue tests (descriptive statistics).

Steel	Cycles to Failure (*N_f_*)							
	Count	Mean	std	min	25%	50%	75%	max
304	32	152,909	101,099	23,929	61,192	152,450	234,882	419,450
316L	32	1,353,132	1,422,401	138,868	418,176	694,903	1,807,082	6,022,466

**Table 5:** Results from the fatigue tests, converted to log scale (descriptive statistics).

Steel	logCycles (dB)							
	Count	Mean	std	min	25%	50%	75%	max
304	32.0	15.35	6.98	1.56	9.71	17.64	21.39	26.43
316	32.0	15.45	8.81	−0.07	9.50	13.68	22.22	32.67

**Table 6:** Main effects, two- and three-factor interactions and *p* values.

Factors	Effect	abs(Effect)	*p*-Value
D	−3.654	3.654	0.084
A	2.984	2.984	0.108
ACD	−2.824	2.824	0.222
AD	2.707	2.707	0.206
AC	1.927	1.927	0.371
BCD	1.885	1.885	0.316
BD	−1.750	1.750	0.417
AB	−1.426	1.426	0.541
CD	−0.886	0.886	0.705
ABD	0.715	0.715	0.742

**Table 7:** Ordinary least square (OLS) regression results.

*β_i_* Effect	*β_i_* Mean	*β_i_* std err	*β_i_* 95% HDI	
			[0.025	0.975]
Intercept	15.4010	0.937	13.524	17.278
D	−1.8272	0.937	−3.704	0.050
A	1.4918	0.937	−0.385	3.369
ACD	−1.4118	0.937	−3.289	0.465
AD	1.3537	0.937	−0.524	3.231
AC	0.9636	0.937	−0.913	2.841
BCD	0.9427	0.937	−0.934	2.820
BD	−0.8748	0.937	−2.752	1.002

**Table 8:** Bayesian regression model summary.

Predictors	Summary Statistics of Posterior								
	Mean	sd	hdi_2.5%	hdi_97.5%	mcse_mean	ess_mean	ess_sd	ess_tail	r_hat
Intercept	15.007	0.991	13.007	16.883	0.012	0.008	7124.0	7105.0	1.0
A	2.074	1.035	−0.013	4.002	0.012	0.009	6936.0	6707.0	1.0
D	−1.964	0.978	−3.935	−0.077	0.011	0.008	8201.0	7225.0	1.0
AD	−0.251	1.117	−2.454	1.914	0.015	0.011	5470.0	5470.0	1.0
AC	1.133	1.000	−0.775	3.147	0.012	0.009	7253.0	6608.0	1.0
BD	−1.403	1.104	−3.508	0.844	0.014	0.010	6169.0	6169.0	1.0
ACD	−1.873	1.084	−3.873	0.352	0.014	0.010	6311.0	6311.0	1.0
BCD	1.545	1.045	−0.461	3.669	0.012	0.009	7049.0	6335.0	1.0
lam	0.063	0.023	0.026	0.109	0.000	0.000	5794.0	4586.0	1.0

**Table 9:** Probability of reaching fatigue life gain of more than 15dB, *p*(*y* > *15*) for all combinations of relief parameters.

Relief Parameters (B, C).	Regime Parameters Values: Force Federate (A, D)			
	(1, 1)	(1, −1)	(−1, 1)	(−1, −1)
(1, 1)	0.21	**0.97**	0.29	0.62
(−1, 1)	0.50	**0.85**	0.62	0.30
(1, −1)	0.55	0.83	0.07	**0.89**
(−1, −1)	**0.82**	0.55	0.26	0.66

## Data Availability

The raw fatigue data and solved Bayesian models can be found and downloaded for free from the GitHub repository: https://github.com/DMDimitrovJ/BurnishingFatigue (accessed on 2 April 2021)
